# Hip Abductor Muscle Strength Recovery: A Comparison Between Joint Replacement Surgery and Internal Fixation Surgery

**DOI:** 10.7759/cureus.59120

**Published:** 2024-04-27

**Authors:** Niketa Patel, Paresh Golwala

**Affiliations:** 1 Department of Physiotherapy, College of Physiotherapy, Sumandeep Vidyapeeth Deemed to be University, Pipariya, Vadodara, IND; 2 Department of Orthopaedics, Sumandeep Vidyapeeth Deemed to be University, Pipariya, Vadodara, IND

**Keywords:** hip abductor muscle strength, hip hemi-arthroplasty, total hip arthroplasty, internal fixation surgeries, fracture neck of femur, intertrochanteric fracture, subtrochanteric fracture

## Abstract

Introduction

Proximal femoral fractures are common fractures of the hip that are considered a major healthcare concern globally; these include subtrochanteric, intertrochanteric, and the neck of the femur fractures. Internal fixation surgery and joint replacement surgery are the two most common intervention techniques used to treat these fractures. Consequently, weakness in the hip abductor muscle post-surgery may lead to implant loosening, necessitating revision of the surgery. In light of this, this study aimed to compare hip abductor strength recovery outcomes between joint replacement surgery and internal fixation surgery.

Methodology

A comparative study was performed over six months at the Department of Orthopaedics and Physiotherapy. Based on the inclusion and exclusion criteria and anticipating potential dropouts, a total of 56 patients were included in the study, and their hip abductor strength was measured using a sphygmomanometer. The patients were classified into two groups: Group A or Group B as per the type of hip surgery. Group A included 29 patients who underwent joint replacement surgeries involving either cemented or uncemented total hip arthroplasty (THA) or hip hemiarthroplasty (HHA). Group B comprised 27 patients who were operated on using either proximal femoral nail (PFN) or dynamic hip screw (DHS).

Results

The cohort consisted of 36 males and 20 females, with a mean age of 51.71 years. The overall mean value of hip abductor muscle strength at postoperative day (POD) three in the internal fixation group was 65.06 ±5.98, which progressed to 107.51 ±24.76 after six months; in the joint replacement surgery group, it was 70.03 ±12.46 at POD three, which progressed to 113.11 ±21.27 after six months. The age-wise distribution demonstrated that the patients in the age group of 18-50 years demonstrated progressive results: from 65.33 ±4.9 at POD three to 105.95 ±22.71 after six months in the internal fixation group; from 66.82 ±7.72 at POD three to 109.59 ±22.54 after six months in the joint replacement group. Moreover, patients aged above 50 years showed progression from 64.80 ±6.98 at POD three to 103.33 ±27.30 after six months in the internal fixation group, and from 69.58 ±14.75 at POD three to 108.22 ±20.62 after six months in the joint replacement group.

Conclusions

Our findings revealed that joint replacement surgery resulted in greater improvements in the hip abductor muscle strength compared to internal fixation surgery in the immediate postoperative period and during follow-ups. Additionally, younger patients exhibited better strength-related outcomes in comparison to the elderly population regardless of the type of surgery.

## Introduction

Hip fractures are considered a global healthcare issue, with an estimated worldwide incidence of 1.6 million per year, which is projected to rise to 2.6 million by 2025 and 4.5 million by 2050. In the Indian population, the annual rate of hip fractures in individuals over the age of 50 years is more than 120 per 100,000, with higher rates observed among women. The most common types of hip fractures are proximal femoral fractures [1,2]. Proximal femur fractures include subtrochanteric, intertrochanteric, and the neck of the femur fractures. Subtrochanteric fracture refers to a fracture occurring between the lesser trochanteric and 5 cm distal to the lesser trochanter [3]. One of the most frequent fractures in older individuals is an intertrochanteric fracture, in which the intertrochanteric area connects the greater and lesser trochanters; it occurs due to low-intensity trauma [4-8]. There are various approaches to treat hip fractures, of which the dynamic hip screw (DHS) is the most commonly used and gold standard approach for intertrochanteric femoral fractures [5,9], while the other common implant used is the proximal femoral nail (PFN) [3,10]. These two approaches are commonly used to fix proximal femur fractures [10].

Total hip arthroplasty (THA) is a standard surgical procedure for avascular necrosis (AVN) of the head of the femur [11] and is performed in hip fracture cases where the bone stock is too poor to form a callus at the fracture site or in the comminuted fracture of the hip joint [12]. Previous studies have provided evidence regarding the success of THA surgery, and its primary goals include pain relief and restoring functions, thereby improving the quality of life of the patients [13-18]. However, a previous experimental study has shown that the weakness in the hip abductor muscle post-THA may lead to implant loosening, necessitating revision of the surgery [13]. Additionally, weak abductor strength post-surgery may result in limping gait and increased risk of falls while severe weakness in hip abductors may result in Trendelenburg gait [19]. Moreover, hip hemiarthroplasty (HHA) is most commonly performed in older patients with neck of the femur fractures, and, similar to THA, HHA could be either cemented or uncemented/cementless [1,20].

While several studies have been conducted to determine the importance of hip abductor muscle strength in THA patients, there exists a dearth of data regarding the comparison of hip abductor strength outcomes between joint replacement and internal fixation surgeries. Hence, this study aimed to compare hip abductor strength recovery after joint replacement surgery and internal fixation surgery.

## Materials and methods

After obtaining approval from the institutional ethical committee (IEC) with reference number COP/596/06/2023, a comparative study was performed over six months at the Department of Orthopaedics and Physiotherapy. The inclusion criteria were as follows: patients operated on for intertrochanteric, subtrochanteric, and fractures of the neck of the femur, THA, HHA, and avascular necrosis of the head of the femur. The exclusion criteria were as follows: individuals with any associated comorbidity that may affect the pain perception, ability to perform active movements and the gait parameters directly, patients with polytrauma, bilateral hip surgeries, and those with a history of previous surgery on either the affected or unaffected hip. The informed consent forms were signed by all the patients willing to participate in the study. All patients were provided with a participation information sheet outlining the important details of the study, such as its benefits, risks, procedure employed, confidentiality measures, and contact details, which helped the participants make informed decisions about whether to participate in the study. The patients were classified into two groups at a 1:1 ratio based on convenient purposive sampling.

Based on the inclusion and exclusion criteria, a total of 60 patients were included in the study. The sample size was calculated using a predetermined level of statistical power set at 80%, with a specified level of significance (p≤0.05). The patients were then placed in either Group A or Group B as per the type of hip surgery. Group A included 30 patients who underwent joint replacement surgeries with either cemented or uncemented THA or HHA. Group B comprised 30 patients who were operated on using either PFN or DHS. The assessor was blinded to the type of surgery to avoid potential bias in the strength measurements. After the allocation, the hip abductor strength was measured using a sphygmomanometer, which is a diagnostic tool commonly used to measure blood pressure [20,21]. A previous study has demonstrated a moderate to high intra-rater reliability with intra-class correlation coefficient (ICC = 0.61 to 0.92) and high inter-rater reliability (ICC = 0.77 to 0.91) along with high concurrent validity (Pearson's r = 0.77 to 0.91) for the application of sphygmomanometer for hip strength assessment [20].

To measure the strength of the hip abductor muscle, the patients were placed in a supine lying position and the sphygmomanometer cuff was inflated to 60 mmHg. It was then positioned between the lateral aspect of the distal femur and the therapist's hand, which provided resistance during the evaluation. After placing the cuff, the patients were instructed to abduct the limb against resistance applied by the therapist. The inflation of the mercury from 60 mmHg was noted. To ensure that the patient understood the instruction of the investigator and for ease of understanding, the hip abductor strength was first measured in the unaffected limb using a sphygmomanometer and then in the affected limb. The patients underwent three trials of each limb. The average value of the trials was considered the final strength of the hip abductor muscles, to ensure reliability and precision as well as to minimize bias. The hip abductor muscle strength was evaluated at four different time points: postoperative day (POD) three, at the time of discharge (POD 12), at the end of six weeks, and the end of six months. When compared to the non-fractured limb, patients generally lose more than 50% of the muscle strength of the fractured limb in the first few weeks postoperatively. Additionally, rehabilitation and recovery kick in after three to four days, leading to an increase in muscle strength and patients gaining control of the limb [22]. However, in Group A, one patient was lost to follow-up, reducing the total number of patients to 29. Similarly, in Group B, three patients were lost to follow-up, bringing the total number of patients down to 27. Hence, the final analysis included 56 patients. 

This comprehensive schedule enabled monitoring and measuring the progression of the hip abductor strength throughout the postoperative recovery period. This detailed methodology outlines the patients’ participation, allocation to the groups, measurement technique, and timings of follow-ups, ensuring clear presentation and encouraging reproducibility of the study. The data were recorded in Microsoft Excel and statistical analysis was performed using SPSS Statistics software (IBM Corp., Armonk, NY). The data were expressed as mean ±standard deviation (SD). A paired t-test was applied for both the groups (cemented or uncemented THA or HHA group and PFN or DHS group) to compare hip abductor muscle strength at POD three and at six months. A p-value less than or equal to 0.05 was considered statistically significant.

## Results

A total of 56 patients were involved in the present study: 36 males and 20 females. The mean age of the patients was 51.71 years. Of the total 56 patients, 12 patients were diagnosed with AVN of femoral head due to sickle cell anemia, long history of steroid intake, and idiopathic conditions, while 44 patients had traumatic hip fractures. Among 12 patients with AVN, two underwent cemented HHA, one was treated with cemented THA, and nine patients were treated with uncemented THA.

Among 44 patients with fractures, one had AVN of the femoral head due to a fracture of the neck of the femur and underwent uncemented THA. Of the remaining 43 patients, 24 suffered intertrochanteric femur fractures, of which eight were treated using DHS, 12 patients were treated using PFN, three underwent cemented THR, and one underwent uncemented THR. Additionally, out of 19 patients that remained, 18 presented with a fracture of the neck of the femur of which three were treated with DHS, three with PFN, one with cemented HHA, four with uncemented HHA, five with cemented THA and two underwent uncemented THA. Only one patient suffered from a subtrochanteric fracture of the femur and was operated on using DHS. The hip abductor muscle strength across different follow-up intervals in joint replacement surgeries vs. internal fixation surgeries is described in Table 1. For the PFN or DHS group, the t-value was 8.94 and the p-value was 0.01, demonstrating statistically significant results when hip abductor muscle strength at POD three was compared with that at six months. Additionally, for cemented or uncemented HHA or THA group patients, the t-value was 10.84 and the p-value was 0.13, demonstrating statistically insignificant results when hip abductor muscle strength at POD three was compared with that at six months.

**Table 1:** Hip adductor muscle strength (mmHg) in patients with joint replacement vs. those with internal fixation surgeries POD: postoperative day; SD: standard deviation

Follow-up interval	Internal fixation surgery (mean ±SD)	Joint replacement surgery (mean ±SD)
POD 3	65.06 ±5.98	70.03 ±12.46
POD 12	72.17 ±11.70	76.92 ±15.97
6 weeks	93.28 ±21.69	97.34 ±17.99
6 months	107.51 ±24.76	113.11 ±21.27

The hip abductor muscle strength in patients aged 18-50 years across different follow-up intervals in terms of joint replacement surgeries vs. internal fixation surgeries is demonstrated in Table 2.

**Table 2:** Hip adductor muscle strength (mmHg) in patients aged 18-50 years with joint replacement versus internal fixation surgeries POD: postoperative day; SD: standard deviation

Follow-up interval	Internal fixation surgery (mean ±SD)	Joint replacement surgery​​​​​​​ (mean ±SD)
POD 3	65.33 ±4.9	66.82 ±7.72
POD 12	76.56 ±11.99	80.05 ±11.84
6 weeks	87.18 ±18.42	92.67 ±17.91
6 months	105.95 ±22.71	109.59 ±22.54

The hip abductor muscle strength in patients aged more than 50 years across different follow-up intervals in terms of joint replacement surgeries vs. internal fixation surgeries is demonstrated in Table 3.

**Table 3:** Hip adductor muscle strength (mmHg) in patients more than 50 years of age with joint replacement versus internal fixation surgeries POD: postoperative day; SD: standard deviation

Follow-up interval	Internal fixation surgery (mean ±SD)	Joint replacement surgery​​​​​​​ (mean ±SD)
POD 3	64.80 ±6.98	69.58 ±14.75
POD 12	70.35 ±10.19	78.73 ±18.19
6 weeks	88.67 ±25.05	93.40 ±18.64
6 months	103.33 ±27.30	108.22 ±20.62

## Discussion

This study compared hip abductor muscle strength outcomes between joint replacement surgery and internal fixation surgery among patients in the age group of 18-50 years and those aged more than 50 years. The postoperative reduction in the hip abductor muscle strength could be attributed to negative consequences of sliding or shortening, damage to the gluteus medius muscle due to shortening (as gluteus medius is shorter than the surrounding iliopsoas, gluteus maximus, and quadriceps muscle), injury to gluteus medius muscle due to the reaming of the greater trochanter for nail insertion, and proximal migration of greater trochanter compromise abductor function impairing patient's ability to walk normally. Therefore, hip abductor muscle strength plays a crucial role after hip surgeries in the phase of rehabilitation [23]. A previous study by Noda et al. concluded that hip muscle strength is an essential outcome measure and should be taken into account by surgeons when deciding the surgical procedure [23]. In this study, the hip abductor strength at POD three, POD 12, at the end of six weeks, and at the end of six months was assessed during the recovery period, which correlated with the study by Lovelock et al., where follow-up post-surgery for various factors involved not only monitoring the progress in the strength post-surgery but also analyzing potential factors leading to failure of the surgery [24].

On comparing the muscle strength between the two groups, a notable difference was observed following both types of surgeries; however, Group A (joint replacement surgery group) showed more improvement throughout the recovery period than Group B (internal fixation surgery group). In Group A, on POD three, the abductor muscle strength was 70.03 mmHg, which significantly (p<0.001) improved to 76.92 mmHg on POD 12, demonstrating improvement during the maximum protection phase post-surgery. Subsequently, the muscle strength significantly improved from POD 12 to six weeks to 97.34 mmHg (p<0.001), demonstrating statistically significant results. Additionally, follow-up at the end of the six months showed an increase in muscle strength to 113.11 mmHg (p<0.001), which was statistically significant. These results displayed sustained improvement and recovery post-surgery.In contrast, Group B patients showed lower muscle strength compared to Group A. In Group B, the muscle strength at POD three was 65.06 mmHg, which improved to 72.17 mmHg at POD 12, which was statistically significant (p<0.001); however, the improvement was less in comparison to Group A. Similarly, the strength improved to 93.28 mmHg at the end of the six weeks with statistically significant results (p<0.001), and, by the end of six months, it improved to 107.51 mmHg (p<0.001).

The results of the present study were found to align with the findings of the study conducted by Noda et al., which compared muscle strength of hip flexors, extensors, and abductors between HHA and gamma nail surgeries in intertrochanteric fractures of the femur and concluded that nailing showed 20-30% decrease in postoperative muscle strength in comparison to HHA [23]. Moreover, a study by Wani et al. examined the effect of THA and internal fixation on hip function during a mean follow-up of 1.5 years among patients with fractures of the neck of the femur and concluded that hip function was generally better in the THA group in comparison to the internal fixation group [25].

The present study further segregated the patients into younger populations (involving those aged 18-50 years) and older patients (more than 50 years of age) to better understand the influence of age and comorbidities on the outcome. The present comparative analysis between younger patients aged 18-50 years in both groups showed the muscle strength of patients in Group A experienced greater improvement in comparison to the patients in Group B, demonstrating a noteworthy difference between the groups. In Group A, the average hip abductor muscle strength of 66.82 mmHg at POD three increased to 109.58 mmHg at six months (p<0.001). Similarly, in Group B, the average muscle strength at POD three was 65.33 mmHg and progressed to 105.95 mmHg (<0.001) at six months, which demonstrated that the improvement in muscle strength was observed but was much lower in comparison to the patients in Group A.

In elderly patients aged more than 50 years, the recovery was markedly low from POD three to POD 12 in Group B. The graph showed a drop in the performance at POD 12 compared to POD three in Group B. Whereas, the patients in Group A showed consistent improvement in muscle strength without any kink in the graph link. Compared to POD 12 values, the patients in Group B recovered to 88.67 mmHg at six weeks and those in Group A recovered to 93.40 mmHg (p<0.001). By the end of the six-month follow-up, Group A recovered to 108.22 mmHg, while Group B reached 103.33 mmHg after six weeks of surgery; hence, while Group B showed rapid improvement in strength, it could not reach the level of Group A.

Our analysis concluded that the younger patients in the age group between 18-50 years had greater improvement in muscle strength compared to the elderly patients, irrespective of the type of surgery they underwent. These findings illustrate that age may play a significant role in muscle strength recovery after hip surgery. Similarly, Létocart et al. investigated muscle adaptation to aging and concluded that aging of the muscles was accompanied by a decrease in muscle mass, consequently leading to a decrease in physical function, which is faster in lower limbs in comparison to the upper limbs [26]. Additionally, recent evidence has shown that age-related decline in lower limb muscle strength affects functional performance in the elderly [27]. These findings emphasize the importance of addressing muscular deficits as part of the intervention for the elderly population. However, we recommend further research focusing on the age-related differences in muscle strength recovery and incorporating this knowledge in customized interventions for the benefit of the patients.

Strength and limitations

The major strength of the study was its finding of a progressive increase in the hip abductor muscle strength in both groups when POD three results were compared to those at 6 months. This study has a few limitations, primarily its small sample size and the limited follow-up period of only six months, which can be extended to one year in future studies. Moreover, the therapists in charge of muscular measurement could not be blinded to the patients’ implants. Also, the potential influence of confounding factors such as gender, comorbidities, and preoperative status on the hip abductor muscle strength in both groups was not evaluated, and these should be addressed in future studies.

## Conclusions

Hip abductor strength plays a pivotal role in the success of hip surgeries. In our study, both types of surgeries led to a progressive increase in hip abductor muscle strength when POD three values were compared to those at six months. However, the joint replacement surgery resulted in greater improvements in the hip abductor muscle strength compared to the internal fixation surgery in the immediate postoperative period and during follow-ups. Additionally, younger patients exhibited better strength outcomes in comparison to the elderly population regardless of the type of surgery. This suggests that joint replacement surgeries may offer distinct advantages in terms of improving hip abductor muscle strength. However, further research is required to explore the factors that resulted in such differences and also to optimize the outcomes for patients of all ages undergoing hip surgeries. Additionally, the potential influence of confounding factors such as gender, comorbidities, and preoperative status should be assessed in future studies on the topic.
